# Data-Driven Cervical Cancer Prediction Model with Outlier Detection and Over-Sampling Methods

**DOI:** 10.3390/s20102809

**Published:** 2020-05-15

**Authors:** Muhammad Fazal Ijaz, Muhammad Attique, Youngdoo Son

**Affiliations:** 1 Department of Industrial and Systems Engineering, Dongguk University-Seoul, Seoul 04620, Korea; fazal@dongguk.edu; 2 Department of Software, Sejong University, Seoul 05006, Korea; attique@sejong.ac.kr

**Keywords:** cancer, artificial intelligence, digital health, machine learning, medical information systems, cervical cancer, imbalanced data analysis, outlier detection

## Abstract

Globally, cervical cancer remains as the foremost prevailing cancer in females. Hence, it is necessary to distinguish the importance of risk factors of cervical cancer to classify potential patients. The present work proposes a cervical cancer prediction model (CCPM) that offers early prediction of cervical cancer using risk factors as inputs. The CCPM first removes outliers by using outlier detection methods such as density-based spatial clustering of applications with noise (DBSCAN) and isolation forest (iForest) and by increasing the number of cases in the dataset in a balanced way, for example, through synthetic minority over-sampling technique (SMOTE) and SMOTE with Tomek link (SMOTETomek). Finally, it employs random forest (RF) as a classifier. Thus, CCPM lies on four scenarios: (1) DBSCAN + SMOTETomek + RF, (2) DBSCAN + SMOTE+ RF, (3) iForest + SMOTETomek + RF, and (4) iForest + SMOTE + RF. A dataset of 858 potential patients was used to validate the performance of the proposed method. We found that combinations of iForest with SMOTE and iForest with SMOTETomek provided better performances than those of DBSCAN with SMOTE and DBSCAN with SMOTETomek. We also observed that RF performed the best among several popular machine learning classifiers. Furthermore, the proposed CCPM showed better accuracy than previously proposed methods for forecasting cervical cancer. In addition, a mobile application that can collect cervical cancer risk factors data and provides results from CCPM is developed for instant and proper action at the initial stage of cervical cancer.

## 1. Introduction

One form of gynecological cancer is cervical cancer. Cervical cancer complications are often associated with the infection of human papillomavirus. It is a common debilitating disease among women worldwide. It is the third most regularly diagnosed cancer (~485,000 cases) and the fourth worldwide driving cause of cancer-related deaths (236,000) each year [1,2]. The main cause of cervical cancer is persistent infection by oncogenic human papillomavirus (HPV). Cervical intraepithelial neoplasia 1–3 and in situ carcinoma are the early manifestations of cervical cancer [3]. Additional factors, including sexually transmitted infections, oral contraceptive use, smoking status, parity, and diet can add to the development of cervical cancer [4]. Generally, patients detected with cervical cancer at initial phases give no noticeable signs or indications that could lead to misdiagnosis [5]. The danger of cervical cancer can be expanded by 2 to 3 times if an HPV-contaminated patient smokes [6]. In case of multiple pregnancies, female HPV-infected patients without pregnancies have lower occurrence of cervical cancer than those with more than one full-term pregnancy [7].

Past research has revealed that magnetic resonance imaging and diffusion-weighted imaging techniques can classify cervical cancer to some degree [8,9]. The absence of doctor skills and confined medical equipment make cervical cancer a main reason for mortality in low-income countries [10]. Additionally, the absence of doctor prowess and confined medical apparatus cause cervical cancer to be a noteworthy reason for death in low-income nations. Because of preventive steps taken by developed nations, the occurrence of cervical cancer worldwide fluctuates meaningfully [11]. Notwithstanding advances in prevention, screening, analysis, and remedy in the course of the past decade, significant local and worldwide discrepancies in cervical cancer findings have led worldwide gynecological cancer societies to publish proof-based management policies to enhance the quality care for patients [12]. The current diagnosis of cervical cancer relies mainly on histological and morphological examinations without well-defined sensitivity and specificity [13].

A recent study by Ghoneim et al. [14] used convolutional neural networks (CNN) and extreme learning machines for cervical cancer classification. The authors used Herlev database. The proposed CNN-ELM-based system achieved 99.5% accuracy in the detection problem (two-class) and 91.2% in the classification problem (seven-class). Chandran and Kumari [15] proposed a gray-level co-occurrence matrix and a probabilistic neural-network-based system, which achieved an accuracy of 92.8%. The images were MRI images, and the database was private. In [16], authors proposed *k*-NN and ANN-based classification systems. The Herlev database was used in the experiments. The k-NNs-based system achieved 88% accuracy, while the ANNs based system obtained 54% accuracy. Gupta et al. [17] designed a multiple back propagation NN-based system. A private database was used, where the image quality was not good. 95.6% accuracy was obtained by the system. Zhang et al. [18], used CNNs develop a system called DeepPap. Using the Herlev database, the system obtained 98.6% accuracy. Bora et al. [19] used CNNs and some additional features for Pap smear image classification. Based on the features, the accuracies varied between 90% and 95%. Adem et al. [20] used a stacked autoencoder with a soft-max layer and achieved an accuracy of 97.25% in the cervical cancer dataset.

In case of cervical cancer, present studies emphasize the development of more precise models instead of the significance of data pre-processing. The outlier detection approach may be used during the pre-processing step to discover discrepancies in data. Accordingly, a good classifier may be generated for better decision-making. Apparently, machine learning (ML) methods are the most useful in predictions. They are widely applied in numerous kinds of cancer studies. Past studies [10,21,22] have used various ML techniques for cervical cancer diagnosis and prediction. However, ML techniques face some challenges, including problems of missing values in dataset, determining precise attributes, removing outliers from the data, distributing class, and attaining results with higher prediction accuracy. Thus, the present work aims to face these challenges. Prior studies have not combined outlier detection and data balancing for cervical cancer prediction. The present work proposes a cervical cancer prediction model (CCPM) by utilizing density-based spatial clustering of applications with noise (DBSCAN) and iForest for outlier detection, with synthetic minority over sampling technique (SMOTE) and SMOTETomek for data balancing, and random forest for cervical cancer prediction based on risk factors [23,24,25]. Hence, the key novelty of the present study is to combine the outlier detection methods, DBSCAN and iForest, the data oversampling methods, SMOTE and SMOTETomek, and random forest classifier for cervical cancer prediction based on risk factors to improve the prediction performance.

The reminder of the paper is organized as follows. Section 2 explains related works on prediction model for cervical cancer, outlier detection methods, over-sampling methods for data balancing, and random forest. Section 3 presents the dataset description with proposed CCPM and evaluation metrics. Section 4 deals with the feature extraction results, DBSCAN and iForest for outlier detection, and SMOTE and SMOTETomek for balancing the dataset along with the results and discussions of four target variables. In Section 5, concluding remarks are presented.

## 2. Related Work

Past research primarily employed a clinical feature-based approach, genetic feature-based approach, and image classification and segmentation to classify and understand cervical cancer’s presence. In case of cervical cancer cell images, a study by Zhang et al. [26] used various machine learning algorithms and matched their segmentation refinement with an artifact-nucleus classifier, for which random forest has revealed the best output. Along with other robust refinement methods, supervised and unsupervised methods were used to distinguish image patches or superpixels from extracted elements, such as Adaboost detectors [27], support vector machine (SVM) [28] or Gauussian mixture models [29]. In a study by Zhao et al. [30] a novel superpixel-based Markov random field (MRF) segmentation was also implemented for non-overlapping cells.

A linear kernel SVM classifier was used by Tareef et al. [31] on superpixels, accompanied by edge enhancement and adaptive thresholding. The results indicated Nuclei Precision: 94.3%; Recall: 92.0%; Dice similarity coefficient (DSC): 0.926. Cytoplasm DSC: 0.914. In one other study, Zhao et al. 2016 [30] used an MRF classifier with a Gap-search algorithm + Automatic labeling map. The findings revealed that Nuclei DSC: 0.93. Cytoplasm DSC: 0.82. In another work by Tareef et al. [28], the authors used SVM classification + Shape based-guided Level Set based on Sparse Coding for overlapping cytoplasm. The results showed that Nuclei Precision: 95%; Recall: 93%; DSC: 0.93, and Cytoplasm DSC: 0.89.

Tseng et al. [32] have reported three classification models of C5.0, SVM, and extreme machine learning to anticipate cervical cancer reoccurrence and to classify the best associated risk factors by utilizing a clinical dataset (e.g., age, radiation therapy, cell type, and tumor size). Their findings indicate that cell type and radiation therapy are two risk factors associated with reoccurrence of cervical cancer. Their findings uncovered that C5.0 had the greatest classification accuracy ratio for all classifiers [32].

Hu et al. [33] have explained a predictive model using multiple logistic regression analysis and artificial neural network to predict the presence of cervical cancer and to identify the maximum risk factors linked with cervical cancer. They used features such as HPV, four genetic factors, and educational level. The experiment recognized that HLA DRB1× 13-2 and HLA DRB1×3-17 alleles were two risk factors of cervical cancer. Such risk factors have been the source of rising cervical cancer risks. Their results indicated that back-substitution fitting of artificial neural network achieved the highest classification accuracy ratio for all classifiers. Sharma [34] has shown a classification model for identifying stages of cervical cancer using C5.0 with different options such as rule sets, boosting, and advanced pruning. Features such as, for instance, clinical diameter, uterine body, renal pelvic, and primary renal carcinoma have been used. Experimental results indicated that C5.0 with advanced pruning achieved the maximum accuracy ratio to identify stages of cervical cancer [34]. Sobar et al. [35] used social science behavior theory to classify the probability of being at risk from cervical cancer by classification methods such as naïve bayes and logistic regression. Their findings indicated that naïve Bayes had better accuracy than logistics regression [35].

Wu and Zhou [10] identified a classification model based on SVM for the diagnosis of cervical cancer. They used recursive feature elimination (RFE) and principle component analysis (PCA) techniques for feature elimination. Their findings revealed that the SVM-PCA had higher accuracy for features selection than SVM-RFE. Although the SVM method can accurately classify cervical cancer data, its high computation cost is a limitation. Recently, Abdoh et al. [22] have used random forest classifier with SMOTE and feature reduction techniques such as RFE and PCA for cervical cancer diagnosis. Their findings revealed that the SMOTE-RF model exceeded the SVM classification technique, similar to the findings of Wu and Zhou [10].

### 2.1. Feature Selection

Feature selection is defined as the method of choosing a subset of relevant features in data that are the most valuable for model construction. It reduces overfitting and training time with improved accuracy [36]. In the present study, we do not need to utilize all features present in the data for making an algorithm work. We can train the algorithm with the features that are indeed more significant to guaranteeing better findings than utilizing all features.

#### Chi-Squared Feature Selection

Chi-squared feature selection is used to infer a feature’s reliance on the class label [37]. It is one of the most frequently used methods for deciding the features that are effective. In Chi-square, a feature’s information value is measured by calculating the chi-square statistical value [38]. Several studies used chi-square as a feature extraction technique, such as in breast cancer [37], Parkinson’s disease using voice signal [39], cancer classification [38], computer-aided diagnosis of Parkinson’s disease [40], and healthcare tweet classification [41]. In Equation (1) given below, c is the number of classes, I is the number of intervals, E_ij_ is the expected number of samples, and A_ij_ is the number of samples of the C class within the j-th interval. The larger the value of χ^2^, the more information the related feature provides.

(1) $$\chi^{2}=\sum_{\begin{array}{c} i=1 \end{array}}^{C}\sum_{j=1}^{I}\frac{{\left(A_{\mathrm{ij}}-E_{\mathrm{ij}}\right)}^{2}}{E_{\mathrm{ij}}}$$

### 2.2. Outlier Detection Method

In information science, an outlier is an observation point that is far off from the bulk of observations. Outlier detection is defined as the way toward identifying and removing outliers from a dataset. The key benefit of removing outliers is that it will improve the accuracy. To the best of our knowledge, none of the studies in cervical cancer used the outlier removal technique. Several studies have reported that by removing the outliers while using the DBSCAN method [42,43,44,45], the performance of the prediction system is improved. Hence, we foresee that the outlier detection methods are able to enhance the accuracy of the classification model for cervical cancer. The present work used two outlier detection techniques, namely DBSCAN and iForest.

#### 2.2.1. DBSCAN

This is a clustering-based method of outlier detection which may be used to isolate outliers [23]. Outliers are points that do not have a place in any cluster. The two key parameters of DBSCAN are epsilon (eps) and minimum points (MinPts). The eps shows the radius of neighborhood about a point x (ξ-neighborhood of x), while MinPts explain the minimum number of neighbors inside the eps radius. DBSCAN is a valuable tool for identifying and removing outliers. Past research has revealed that DBSCAN can successfully recognize outliers, showing excellent performance in social network community [43], wireless sensor networks [44], and type 2 diabetes and hypertension [46]. Verbiest et al. [47] have reported that combining outlier removal and oversampling method can produce better outcomes. Accordingly, merging DBSCAN for outlier detection with SMOTE and SMOTETomek methods might improve the accuracy of CCPM.

#### 2.2.2. iForest

Isolation forest (iForest) is an outlier detection technique [48,49]. It differentiates outliers through developing isolation tress (iTrees) and handling outliers as instances/points that have a short average length inside iTrees. Past studies have revealed noteworthy findings using iForest for outlier and anomaly detection. Domingues et al. [50] have estimated diverse outlier detection techniques by means of UCI repository datasets. Their findings revealed that iForest could be successfully used to classify outliers while providing outstanding scalability on large datasets with bearable memory use. Calheiros et al. [51] have applied iForest for unsupervised anomaly detection to locate concerns in large scale cloud datacenters. Their findings indicate that iForest can be viable and beneficial to locate the anomaly. iForest uses the property that outliers are more vulnerable to isolation, so it is possible to identify outliers as observations with short predicted track lengths (i.e., less splits) throughout the forest [52]. Many studies used iForest as an outlier detection technique such as in the detection of insulin pump in artificial pancreas [53], fault detection in artificial pancreas [54], medication errors [55], diabetics [56], Medicare provider fraud [52], and detection of anomalous vital signs of the elderly [57].

### 2.3. Oversampling Method for Imbalance Dataset

Machine learning methods can confront difficulties when one class dominates a dataset (i.e., the number of records in one class exceeds the number of other classes by very much). This dataset is called an imbalanced dataset. It deceives the classification, with a negative impact on findings. In the present study, we used SMOTE and SMOTETomek to handle the imbalanced dataset problem.

#### 2.3.1. SMOTE

SMOTE is a technique of oversampling proposed by Chawla et al. [24]. This randomly produces a new minority class instances from the sample’s nearest minority class neighbors. These instances are created taking into account features of the original dataset, with the objective that they conclude original minority class instances. It is used in various fields including breast cancer detection [58,59], liver cancer [60], and cervical cancer [22] to resolve the unbalanced problem. To increase the minority class, SMOTE uses Equation (2)

(2) $$x_{syn}=x_{i}+(x_{knn}-x_{i})\times t$$

Firstly, SMOTE recognizes the feature vector $x_{i}$ and find the K-nearest neighbors $x_{knn}$. Then, it calculates the difference between the feature vector and k-nearest neighbor. Thereafter, it multiplies the difference by a random number from 0 to 1. It then adds the output number to feature vector to identify a new point on the line segment. Lastly, it repeats the above steps to find feature vectors.

#### 2.3.2. SMOTETomek

SMOTETomek is a technique used to handle imbalanced data. Many past studies have used SMOTETomek and revealed favorable outcomes in balancing the data and enhancing the model performance. It showed better area under the curve value than synthetic minority oversampling technique edited nearest neighbor (SMOTEENN) when numerous imbalanced datasets are used [61]. Goel et al. [62] have reviewed five sampling techniques to resolve the imbalanced data problem by using eight datasets from the UCI repository. Their findings indicate that for most datasets, SMOTETomek can increase the model accuracy. Chen et al. [63] have used SMOTETomek to resolve the imbalanced data issue in lane-changing behavior and random forest to foresee the risk associated with lane changing. Their result revealed that SMOTETomek considerably enhanced the model by as much as 80.3%. Tomek Links can be described as a method for undersampling or as a technique for cleaning up data. They can be identified as a pair of the nearest neighbors of opposite classes, which are minimally distant [64]. They are used to remove the overlapping samples that SMOTE adds. Past studies used SMOTETomek as oversampling technique in various healthcare areas such as self-care problem identification for children with disability [65], cancer gene expression data [66], vertebral column pathologies, diabetes and Parkinson’s disease [67], and breast cancer [68].

### 2.4. Random Forest

RF algorithm is an ensemble classifier which generates multiple decision trees along with weak classifiers learned from the data on a random sample [69,70]. RFs vanquish numerous issues with decision trees. For instance, they can reduce overfitting and produce low variance. We used random forest for prediction in cervical cancer. The following steps describe the generation of each tree in random forest:

- Choose a value of n that shows the number of trees that will be increased in a forest;
- Generate n bootstrap samples with bagging technique of the training set;
- For each bootstrap dataset, grow a tree. If this training set would consist of M number of input variables, m<<M number of inputs are selected randomly out of M and the best split on these m attributes is used to split the node. The value of m will remain constant during forest growing;
- The tree will be grown to the largest possible level;
- The prediction results are obtained from the model (most frequent class) of each decision tree in the forest.

Past work has revealed that RF is useful for predicting cervical cancer, with high classification accuracy [22]. Past studies have shown that the outlier data, together with imbalanced datasets, are difficult issues in classification. For instance, they may decrease the system’s overall performance [47]. Hereafter, we proposed a CCPM that comprised DBSCAN and iForest for outlier detection to eliminate the outlier data, SMOTE and SMOTETomek for class balancing, and RF for predicting cervical cancer. By eliminating outlier data along and balancing the dataset, RF is expected to give better results.

## 3. Dataset Description

The used dataset was published on the repository of UCI collected at Hospital Universitario de Caracas in Caracas, Venezuela [71]. The dataset contained 858 instances with 36 features. Table 1 displays dataset features, total number of entries, and the missing value for each feature. To deal with missing values, the present study used the mean equation as depicted in Equation (3).

(3) $$\bar{x}=\frac{1}{n}\left(\sum_{i=1}^{n}x_{i}\right)=\frac{x_{1}+x_{2}+x_{3}+x_{4}+\cdots }{n}$$

There are four target variables (Schiller, Hinselmann, Cytology, and Biopsy). Schiller’ test uses iodine solution in cervix. The cervix is examined by naked eye to diagnose cervical cancer [72]. Due to its poor performance, the Schiller’ test has been replaced by cytology. Cytology test is used to examine cancer, precancerous conditions, and urinary tract infection. Hinselmann’s test is applied to study the cervix, vulva, and vagina.

### 3.1. Prediction Model for Cervical Cancer

The proposed CCPM consists of outlier detection based on DBSCAN and iForest. It also has SMOTE and SMOTETomek to balance the data with RF for cancer prediction.

**Figure 1:**
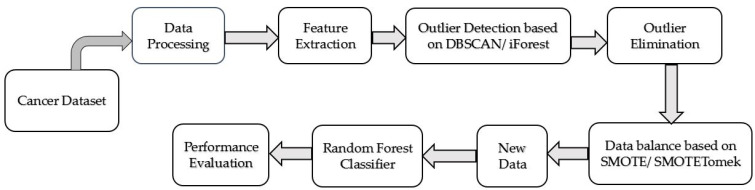
Prediction model for cervical cancer.

Lastly, the performance of the proposed CCPM is compared with the performances of other existing models. Figure 1 elucidates the proposed CCPM model.

We used 70 % dataset values for training and 30% for testing with 10 cross validation. We have used the Python programming language and scikit-learn, pandas, numpy libraries used for machine learning models. For outlier detection, we have used scikit-learn library in python programming language [73]. For oversampling, we have used the imbalanced-learn Python library [74].

### 3.2. Evaluation Metrics

The prediction output may have the following four possible outcomes on the basis of a confusion matrix [75], true positive (TP), true negative (TN), false positive (FP), and false negative (FN). Table 2 displays the precision, recall, specificity, F1 score, and accuracy. Table 3 shows different outcomes of two-class prediction.

## 4. Results and Discussion

This section deals with the results of feature extraction and four scenarios of CCPM in terms of precision, sensitivity, specificity, F1 score, and accuracy. Four scenarios are divided into four sections. Each section displays results and their explanation. We then compared biopsy results with results of past studies and some practical implications to conclude this section.

### 4.1. Feature Extraction Results

In the present study, we used chi-square to extract the features from the dataset. The main aim of the feature extraction technique is to extract the most valuable features from a given rather than the whole features. For simplicity, we have extracted first ten features that have the highest chi-score. Besides, we have added the chi-score of ten variables in the feature extraction table. We used these features for our analysis. After selecting these features, we used outlier detection techniques to remove outliers from the data. Table 4 displays the results of chi-square.

### 4.2. DBSCAN and iForest for Outlier Detection

**Figure 2:**
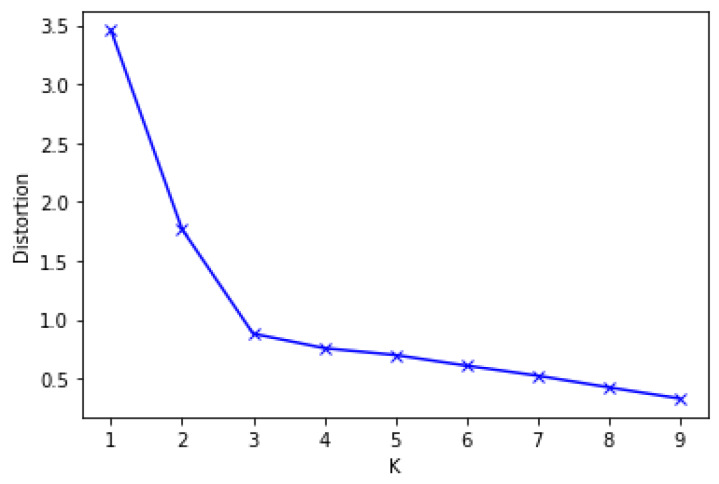
Optimal eps value for DBSCAN.

To implement the DBSCAN-based outlier detection, the optimum value of MinPts and eps must first be established. If the value of eps is too low, it will generate more clusters and normal data may be counted as outliers. On the other hand, if it is too large, it will produce fewer clusters, and true outliers could be categorized as normal data [23,43,44]. We specified the MinPts value to be 5. Next, we have to determine the optimal number of eps. First, we measure each point’s average distance from its nearest neighbors. The value k represents MinPts and is outlined by the user. The goal is to decide the “knee” used to estimate the parameter collection of eps. A “knee” is the point at which a sharp shift occurs along the k-distance curve [46]. Figure 2 displays the k-dist graph sorted for the cervical cancer data set and the optimal value of eps. The "knee" shows up at the distance of 3 in the cervical cancer dataset. Lastly, the outlier data are excluded, and standard data are used for further analysis.

iForest works in two phases. The first (training) stage constructs isolation trees using training set subsamples. The second stage (testing) passes through isolation trees to obtain an outlier score for each case. Both subsample size (MaxSample) and number of trees to be built (NumTree) are essential parameters to be calculated. The iForest works well when the MaxSample is kept small; the larger MaxSample reduces the ability of iForest to isolate outer data, as normal data can meddle with isolation [48,49,50]. Number of Trees influences the scale of the ensemble. We tried different configuration parameters and found that MaxSample is 10% of the total data size and NumTree is 100% optimal. The iForest was implemented using scikit-learn python library. In case of DBSCAN, we found two outliers. We removed these outliers and processed the data for further analysis. However, iForest found eighty-six outliers. We removed those outliers as well and processed the data.

### 4.3. SMOTE and SMOTETomek for Balancing the Dataset

We also used over-sampling methods to increase the number of cases in a balanced way. We applied SMOTE or SMOTETomek methods to balance the datasets. SMOTE oversamples the minority class to randomly generate instances and increase minority class instances, and Tomek under-samples a class to remove noise while maintaining balanced distributions. As can be seen in Table 5, the dataset is balanced after the application of SMOTE and SMOTETomek. The classification aim is to diminish errors during the learning process; hence, we anticipate that a better model accuracy can be attained from the balanced datasets.

### 4.4. Results of Target Variables: Biopsy, Schiller, Hinselmann, Cytology

The ten features extracted by Chi-square were used for all models (SVM, multilayer perceptron (MLP), logistic regression (LR), naïve Bayes, and K-nearest neighbors (KNN)), and all four target variables (Biopsy, Schiller, Hinselmann, and Cytology). For each of the target variables, the CCPM was compared with other conventional machine learning models. The results of CCPM for each of the target variables outperformed previous machine learning approaches (with reference to Table 6, Table 7, Table 8, Table 9, Table 10, Table 11, Table 12, Table 13, Table 14, Table 15, Table 16, Table 17, Table 18, Table 19, Table 20 and Table 21). The key reason for these results is the combination of the outlier removal and data balancing techniques. Hence, this improves the accuracy for our CCPM.

### 4.5. Comparison with Previous Studies

We compared the results of our CCPM with past studies that used the cervical cancer dataset. The study by Wu and Zhu [10] used SVM-RFE and SVM-PCA, while Abdoh et al. [22] used SMOTE-RF-RFE and SMOTE-RF-PCA. Table 22, Table 23, Table 24 and Table 25 illustrates the comparison of results on the sensitivity, specificity, and accuracy of all target variables of CCPM with Wu and Zhu [10] and Abdoh et al. [22]. All four proposed scenarios of CCPM surpassed past studies by Wu and Zhu [10] and Abdoh et al. [22] in terms of sensitivity, specificity, and accuracy. A study by Deng et al. [76] used SMOTE for handling the imbalanced data, and SVM, XGBoost and RF to identify the risk factors of cervical cancers. Our CCPM produces better results as compared to the Deng et al. [76]. They accuracies achieved by SVM, XGBoostand Random Forest are 90.34, 96.34, and 97.39, respectively. A recent study by Adem et al. [20] used a stacked autoencoder with a soft-max layer and achieved an accuracy of 97.25% in the cervical cancer dataset. Our CCPM surpassed Deng et al. [76] and Adem et al. [20] in terms of accuracy. Thus, we can conclude that our proposed CCPM is better than other machine learning models as well as past studies.

Our proposed CCPM has four combinations, two outlier detection methods and two data oversampling methods, and their results varied target variables and performance measures. For example, the combination of iForest and SMOTETomek showed the best performances in sensitivity and accuracy in biopsy and specificity in Schiller and Hinselmann, but that of DBSCAN and SMOTE was the best in sensitivity and accuracy in Schiller and Hinselmann tests. In summary, iForest showed better results than DBSCAN for biopsy tests, but DBSCAN was usually better than iForest for the cytology test. However, we recommend using all of four combinations for predicting cervical cancer and using ensemble results of them for more robust prediction.

In case of risk factors, Table 1 showed the entire attributes’ name and the corresponding number of each attribute. The ten features in our study were Smokes (years), Hormonal Contraceptives (years), STDs (number), STDs: genital herpes, STDs: HIV, STDs: Number of diagnosis, Dx: Cancer, Dx: HPV, Dx. We compared the results of risk factors based on feature extraction results with past studies [10,22,77]. The factor Dx: Cancer found in our study is also validated by the Wu and Zhu [10], while the factors smokes per years and Hormonal contraceptives found by Wu and Zhu [10], and Smokes and Hormonal Contraceptives found by Abdoh et al. [22] seemed quite relevant to the factors Smokes (years) and Hormonal Contraceptives (years) factors found in the present study. Furthermore, we compared our features result with past study by Nithya and Ilango [77]. These findings have revealed that the common features between both studies include Smokes (years), Hormonal Contraceptives (years), STDs: Number of diagnosis, Dx: Cancer, Dx: HPV, and Dx. Hence, Smokes (years), Hormonal Contraceptives (years), STDs: Number of diagnosis, Dx: Cancer, Dx: HPV, and Dx are the factors that corresponds to the riskiest factors of cervical cancer. Based on our experiment the existence of these factors enhances the risk of being a cervical cancer patient. The present work identified the topmost significant features for the classification of cervical cancer patients to be Smokes (years), Hormonal Contraceptives (years), STDs: Number of diagnosis, Dx: Cancer, Dx: HPV, and Dx.

The complexity of an algorithm is generally calculated using Big-O notation [78,79,80]. Time complexity and space complexity are two types of computational complexity [81,82]. Time complexity deals with how long the algorithm is executed for, while space complexity deals with how much memory is used by its algorithm. An algorithm will process amounts of data, where N is a symbol of amounts of data. If an algorithm does not depend on N, then the algorithm has constant complexity or symbolized by O(1) (Big-O one). On the contrary, if the algorithm is dependent on N, the complexity depends on line code in algorithm and it is can be O(*n*), O(*n^2^*), O(log *n*) and others. Let *n* be the number of training examples, *d* be the number of dimensions of the data, *k* be the number of neighbors or the number of trees, and *c* be the number of classes. Table 26 depicts the time and space complexities of some machine learning algorithms. In our CCPM, the RF becomes slow and requires more memory space for training as compared to other algorithms. In addition, the proposed CCPM requires additional computation for outlier detection and data balancing. In our CCPM, however, we got better accuracy compared to other conventional machine learning methods.

### 4.6. Practical Implications

Lee et al. [83] have studied the impacts of educational text messages concerning HPV vaccination and its advantages and observed a substantial upsurge in HPV vaccination intake in targeted populations. Cancer screening programs have also used text messaging in an attempt to tackle the screening intake. Weaver et al. [84] have recently examined how elderly patients would be interested in text messages intended to motivate their participation in a screening program. Their findings indicated that older populations were extremely interested in such messages based on their involvement. A recent study by Ijaz et al. [46] has used IoT for a healthcare monitoring system for patients at home and used personal healthcare devices that perceive and estimate a persons’ biomedical signals. The system can notify health personnel in real-time when patients experience emergency situations. 

**Figure 3:**
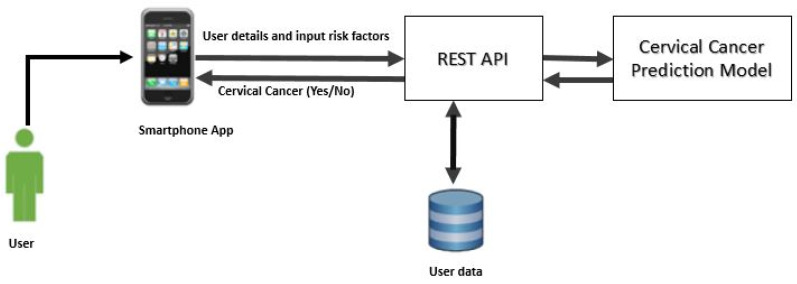
Cervical Cancer Predication Model architecture framework.

In the present work, we implemented CCPM into a mobile app to show its practical implication for simple users. Figure 3 shows the architecture framework of CCPM. A mobile app collects user’s risk factor data and then sends it to Representational State Transfer (REST API) to be stored in a a secure remote server. We used NoSQL MongoDB to store user data by keeping in mind that it could store large amounts of data. Finally, CCPM was used to forecast the presence of cervical cancer as long as users input risk factors. Predication results are shown in the mobile app.

**Figure 4:**
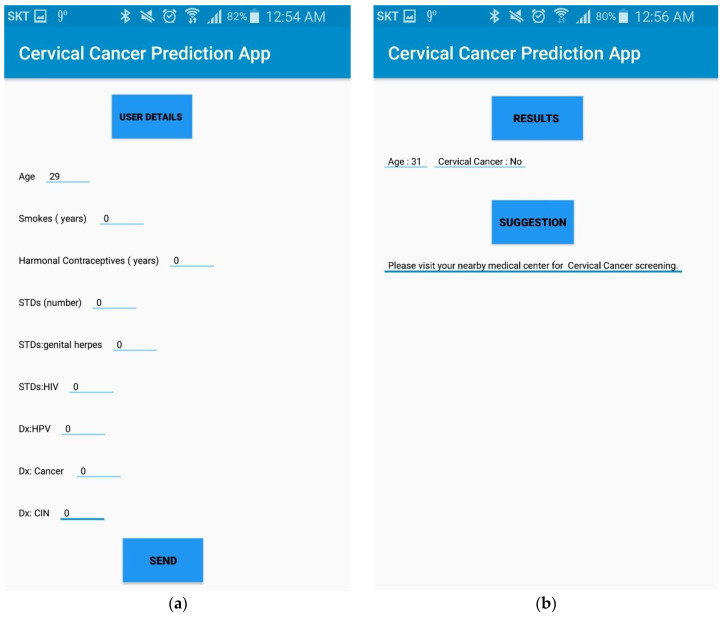
(**a**) Interface of mobile application to gather user’s data. (**b**) Prediction result interface of CCPM mobile application.

Figure 4a,b shows a prototype of the mobile app. When a user presses the “send” button, risk factors that the user inputs are stored in the remote server. CCPM is then activated to foresee the existence of cervical cancer. Figure 4b depicts the interface of mobile application when the user receives prediction results. Therefore, it is expected that the CCPM mobile application can help users find the risk of cervical cancer proficiently at an early stage.

In our CCPM mobile application, maintaining the safety of healthcare data is of paramount importance. The data in the CCPM mobile application are encrypted. Encryption is an essential for our CCPM mobile application since it scrambles a user’s personal data. We used secure sockets layer (SSL) technology that encrypts information transmitted between a mobile application and the server. SSL uses a cryptographic system that uses two keys to encrypt data, e.g., a public key known to everyone and a private or secret key known only to the recipient of the message [85,86]. The NoSQL MongoDB helps to store the user data. A secure log-in feature can be added to mobile application which is called two-factor authentication (2FA). 2FA improves the mobile application’s security. Some examples of 2FA include: Username/password + SMS code, Username/password + code sent via email, and Username/password + biometric authentication (a fingerprint). Another important feature to secure the user data is with the help of data wiping. To implement data wiping, an app can: log out a user after a certain period of inactivity, keep information in an encrypted form, and perform an automatic data wipe after a certain number of unsuccessful login attempts. This gives users a sense of greater control over privacy, security, and confidentiality in towards their healthcare data. The security systems described above can keep the health data secured and safe in our mobile application.

## 5. Conclusions

As indicated by the World Health Organization (WHO), about 80% cases of cervical cancer are noted in developing nations. A cure ratio is described as the ratio of female cases that are healed from the disease. It can be boosted by classifying the risk factors of cervical cancer [22]. This study proposed a CCPM that used Chi-square as feature extraction technique. We extracted ten features and used them in our study. The current dataset is unbalanced. It has a lot of missing values. For missing values, we used mean equation. The current work proposed CCPM by joining DBSCAN and iForest for outlier detection, with SMOTE and SMOTETomek for class balancing and RF as a classifier. The CCPM can help users find the risk of cervical cancer at an early stage. Accuracies achieved by Biopsy forDBSCAN + SMOTETomek + RF, DBSCAN + SMOTE+ RF, iForest + SMOTETomek + RF, and iForest + SMOTE + RF were 96.708%, 97.007%, 98.919%, and 98.925%, respectively. While for Schiller accuracies for DBSCAN + SMOTETomek + RF, DBSCAN + SMOTE+ RF, iForest + SMOTETomek + RF, iForest + SMOTE + RF were 99.48%, 99.22%, 98.50%, and 98.71% respectively. In case of Hinselmann, the accuracies achieved by DBSCAN + SMOTETomek + RF, DBSCAN + SMOTE+ RF, iForest + SMOTETomek + RF, iForest + SMOTE + RF were 99.50%, 99.01%, 99.50%, and 99.50% respectively. For Cytology, the accuracies achieved by DBSCAN + SMOTETomek + RF, DBSCAN + SMOTE+ RF, iForest + SMOTETomek + RF, iForest + SMOTE + RF were 97.72%, 97.22%, 97.50%, and 97.51% respectively. Hence, combining iForest with SMOTE and SMOTETomek can produce better results than combining DBSCAN with SMOTE and SMOTETomek. Besides, we compared Hinselmann, Schiller, Cytology, and Biopsy results with past studies by Wu and Zhu [10] and Abdoh et al. [22] in terms of sensitivity, specificity, and accuracy. Our results revealed that DBSCAN + SMOTETomek + RF, DBSCAN + SMOTE+ RF, iForest + SMOTETomek + RF, iForest + SMOTE + RF surpassed past studies by Wu and Zhu [10] and Abdoh et al. [22]. Besides, Our CCPM surpassed Deng et al. [76] and Adem et al. [20] in terms of accuracy. As a result, we can conclude that our proposed CCPM is better than other models as well as past studies.

In future work, we will employ more diverse techniques for outlier detection and over-sampling methods. We will also apply each combination to CCPM to improve its diagnosis performance. The proposed method can be applied to other cervical cancer datasets. Results on these may provide additional intuitions for the early diagnosis of cervical cancer.

This study also has a limitation, as only one dataset is employed. Since we only focus on cervical cancer in this study, we only used one dataset. In future research, the proposed CCPM can be applied to diverse cancer datasets (such as breast, liver, lung, prostate, thyroid, and kidney) to enhance the clarity and quality of results. Another limitation is that our algorithm (which is a combination of outlier technique and data balancing with RF) becomes slower and needs more memory to run, but as we got a better accuracy, it serves our purpose.

## Figures and Tables

**Table 1:** Dataset features, number of entries, and missing values.

Number	Attribute Name	Type	Missing Values
1	Age	Int	0
2	Number of sexual partners	Int	26
3	First sexual intercourse (age)	Int	7
4	Num of pregnancies	Int	56
5	Smokes	bool	13
6	Smokes (years)	bool	13
7	(Smokes (packs/year)	bool	13
8	Hormonal Contraceptives	bool	108
9	Hormonal Contraceptives (years)	Int	108
10	Intrauterine Device (IUD)	bool	117
11	IUD (years)	Int	117
12	Sexually Transmitted Disease (STD)	bool	105
13	STDs (number)	Int	105
14	STDs: condylomatosis	bool	105
15	STDs: cervical condylomatosis	bool	105
16	STDs: vaginal condylomatosis	bool	105
17	STDs: vulvo-perineal condylomatosis	bool	105
18	STDs: syphilis	bool	105
19	STDs: pelvic inflammatory disease	bool	105
20	STDs: genital herpes	bool	105
21	STDs: molluscum contagiosum	bool	105
22	STDs: AIDS	bool	105
23	STDs: HIV	bool	105
24	STDs: Hepatitis B	bool	105
25	STDs: HPV	bool	105
26	STDs: Number of diagnosis	Int	0
27	STDs: Time since first diagnosis	Int	787
28	STDs: Time since last diagnosis	Int	787
29	Dx: Cancer	bool	0
30	Dx: Cervical Intraepithelial Neoplasia (CIN)	bool	0
31	Dx: Human Papillomavirus (HPV)	bool	0
32	Diagnosis: Dx	bool	0
33	Hinselmann: target variable	bool	
34	Schiller: target variable	bool	
35	Cytology: target variable	bool	
36	Biopsy: target variable	bool	

**Table 2:** Performance metrics for the classification model.

Performance Metric	Formula
Precision	TP/(TP+FP)
Recall/Sensitivity	TP/(TP+FN)
Specificity/True Negative Rate	TN/(TN + FP)
F1 Score	2 × (Precision × Recall)/(Precision + Recall)
Accuracy	(TP+TN)/(TP+TN+FP+FN)

**Table 3:** Different outcomes of two-class prediction.

	Predicted as “Yes”	Predicted as “No”
Actual “Yes”	True Positive (TP)	False Negative (FN)
Actual “No”	False Positive (FP)	True Negative (TN)

**Table 4:** Results of Chi-square.

No	Feature’s Name	Features Scores
1	Smokes (years)	421.4689
2	Hormonal Contraceptives (years)	246.6243
3	Sexually Transmitted Diseases (STDs) (number)	87.28867
4	STDs: genital herpes	43.73654
5	STDs: HIV	29.35086
6	STDs: Number of diagnosis	21.74795
7	Dx: Cancer	21.74795
8	Dx: cervical intraepithelial neoplasia (CIN)	20.71644
9	Dx: human papillomavirus (HPV)	12.64184
10	Dx	12.44904

**Table 5:** Results of synthetic minority over sampling technique (SMOTE) and SMOTETomek.

Before SMOTE		After SMOTE		Before SMOTETomek		After SMOTETomek	
Minority (%)	Majority (%)	Minority (%)	Majority (%)	Minority (%)	Majority (%)	Minority (%)	Majority (%)
55 (6.41%)	803 (93.59%)	803 (93.59%)	803 (93.59%)	55 (6.41%)	803 (93.59%)	803 (93.59%)	803 (93.59%)

**Table 6:** Performance evaluation results based on density-based spatial clustering of applications with noise (DBSCAN) and SMOTE for Biopsy.

Method	Precision (%)	Recall/Sensitivity (%)	Specificity (%)	F1 Score (%)	Accuracy (%)
SVM	92.797	91.666	93.908	92.768	92.768
MLP	96.049	97.549	94.416	96.000	96.001
Logistic Regression	94.020	93.627	94.416	94.015	94.014
Naïve Bayes	93.666	96.568	90.355	93.506	93.516
KNN	94.289	98.039	89.847	94.001	94.014
Proposed CCPM (Random Forest)	97.025	98.039	95.939	97.006	97.007

**Table 7:** Performance evaluation results based on DBSCAN and SMOTETomek for Biopsy.

Method	Precision (%)	Recall/Sensitivity (%)	Specificity (%)	F1 Score (%)	Accuracy (%)
SVM	94.692	93.782	95.544	94.682	94.683
MLP	95.697	95.854	95.544	95.696	95.696
Regression	95.697	95.854	95.544	95.696	95.696
Naïve Bayes	93.587	96.373	90.594	93.416	93.417
KNN	94.430	94.300	94.554	94.430	94.430
Proposed CCPM (Random Forest)	96.720	97.409	96.039	96.720	96.708

**Table 8:** Performance evaluation results based on iForest and SMOTE for Biopsy.

Method	Precision (%)	Recall/Sensitivity (%)	Specificity (%)	F1 Score (%)	Accuracy(%)
SVM	95.501	90.957	99.456	95.154	95.161
MLP	96.432	93.085	99.456	96.233	96.236
Regression	96.131	93.085	98.913	95.965	95.967
Naïve Bayes	95.656	92.021	98.913	95.426	95.430
KNN	98.668	97.872	99.456	98.655	98.655
Proposed CCPM (Random Forest)	98.924	98.936	98.130	98.924	98.925

**Table 9:** Performance evaluation results based on iForest and SMOTETomek for Biopsy.

Method	Precision (%)	Recall/Sensitivity (%)	Specificity (%)	F1 Score (%)	Accuracy (%)
SVM	94.726	91.935	97.282	94.591	94.594
MLP	96.845	94.623	98.913	96.755	96.756
Logistic Regression	94.853	90.860	98.369	94.587	94.594
Naïve Bayes	94.619	90.322	98.369	94.316	94.324
KNN	97.302	96.774	97.826	97.297	97.297
Proposed CCPM (Random Forest)	98.918	98.924	98.913	98.918	98.919

**Table 10:** Performance evaluation results based on DBSCAN and SMOTE for Schiller.

Method	Precision (%)	Recall/Sensitivity (%)	Specificity (%)	F1 Score (%)	Accuracy (%)
SVM	95.582	96.292	94.807	95.572	95.572
MLP	97.759	96.825	95.710	96.759	97.662
Logistic Regression	95.297	91.594	98.498	95.096	95.106
Naïve Bayes	93.165	90.963	96.033	93.589	93.575
KNN	92.205	93.440	91.119	92.247	92.244
Proposed CCPM (Random Forest)	98.216	99.208	99.487	99.217	99.217

**Table 11:** Performance evaluation results based on DBSCAN and SMOTETomek for Schiller.

Method	Precision (%)	Recall/Sensitivity (%)	Specificity (%)	F1 Score (%)	Accuracy (%)
SVM	95.393	93.298	97.368	95.393	95.393
MLP	97.912	97.883	97.938	97.762	97.911
Regression	93.641	87.891	94.680	93.188	93.105
Naïve Bayes	93.587	96.373	90.594	93.416	93.417
KNN	94.580	97.387	91.150	94.261	91.260
Proposed CCPM (Random Forest)	99.509	99.484	99.463	99.474	99.479

**Table 12:** Performance evaluation results based on iForest and SMOTE for Schiller.

Method	Precision (%)	Recall/Sensitivity (%)	Specificity (%)	F1 Score (%)	Accuracy (%)
SVM	96.613	94.623	97.563	96.141	96.143
MLP	98.771	97.291	98.677	98.774	97.724
Regression	94.497	91.714	96.938	94.363	94.373
Naïve Bayes	93.048	92.746	93.343	93.098	93.098
KNN	93.317	94.514	91.344	93.881	93.881
Proposed CCPM (Random Forest)	98.714	97.314	100.00	98.714	98.714

**Table 13:** Performance evaluation results based on iForest and SMOTETomek for Schiller.

Method	Precision (%)	Recall/Sensitivity (%)	Specificity (%)	F1 Score (%)	Accuracy (%)
SVM	94.011	93.625	94.329	94.054	94.010
MLP	97.369	96.808	96.913	97.509	97.164
Logistic Regression	94.142	91.635	96.681	94.073	94.072
Naïve Bayes	93.085	92.000	95.172	93.866	93.866
KNN	94.762	94.707	91.344	93.072	93.072
Proposed CCPM (Random Forest)	98.463	98.907	98.074	98.499	98.495

**Table 14:** Performance evaluation results based on DBSCAN and SMOTE for Hinselmann.

Method	Precision (%)	Recall/Sensitivity (%)	Specificity (%)	F1 Score (%)	Accuracy (%)
SVM	98.500	98.492	98.507	98.500	98.500
MLP	98.759	100.00	98.760	98.759	98.759
Logistic Regression	97.796	98.994	96.568	97.766	97.766
Naïve Bayes	97.165	98.963	95.433	97.089	97.087
KNN	96.905	98.440	95.433	96.847	96.844
Proposed CCPM (Random Forest)	99.016	100.00	97.948	98.997	98.997

**Table 15:** Performance evaluation results based on DBSCAN and SMOTETomek for Hinselmann.

Method	Precision (%)	Recall/Sensitivity (%)	Specificity (%)	F1 Score (%)	Accuracy (%)
SVM	99.004	99.512	98.461	98.999	98.053
MLP	98.792	100.00	97.512	98.762	98.620
Regression	98.034	98.989	97.073	98.015	98.015
Naïve Bayes	93.587	96.373	90.594	93.416	93.417
KNN	97.580	98.507	96.550	97.561	97.560
Proposed CCPM (Random Forest)	99.509	100.00	98.963	99.504	99.504

**Table 16:** Performance evaluation results based on iForest and SMOTE for Hinselmann.

Method	Precision (%)	Recall/Sensitivity (%)	Specificity (%)	F1 Score (%)	Accuracy (%)
SVM	99.514	97.012	97.024	98.509	98.530
MLP	98.771	96.050	98.677	98.774	98.270
Regression	98.537	97.014	98.058	98.533	98.533
Naïve Bayes	98.048	98.238	96.172	98.048	98.048
KNN	98.317	99.514	97.044	98.288	98.288
Proposed CCPM (Random Forest)	99.514	99.014	100.00	99.504	99.504

**Table 17:** Performance evaluation results based on iForest and SMOTETomek for Hinselmann.

Method	Precision (%)	Recall/Sensitivity (%)	Specificity (%)	F1 Score (%)	Accuracy (%)
SVM	97.755	96.431	99.481	99.754	98.754
MLP	97.369	97.326	98.913	99.509	98.509
Logistic Regression	98.529	99.065	97.927	98.525	98.525
Naïve Bayes	97.085	98.000	96.172	97.066	97.066
KNN	97.782	98.507	97.044	97.772	97.772
Proposed CCPM (Random Forest)	98.514	100.00	98.974	99.509	99.509

**Table 18:** Performance evaluation results based on DBSCAN and SMOTE for Cytology.

Method	Precision (%)	Recall/Sensitivity (%)	Specificity (%)	F1 Score (%)	Accuracy (%)
SVM	94.475	99.519	87.807	93.852	93.872
MLP	91.759	90.465	92.710	94.759	94.682
Logistic Regression	84.999	80.000	89.393	84.637	84.635
Naïve Bayes	80.655	71.065	88.345	79.792	79.900
KNN	94.002	99.000	87.878	93.467	93.521
Proposed CCPM (Random Forest)	97.225	96.428	97.989	97.215	97.217

**Table 19:** Performance evaluation results based on DBSCAN and SMOTETomek for Cytology.

Method	Precision (%)	Recall/Sensitivity (%)	Specificity (%)	F1 Score (%)	Accuracy (%)
SVM	94.413	98.098	90.000	94.165	94.187
MLP	99.452	99.083	91.052	95.182	95.175
Regression	86.326	77.860	93.137	85.490	85.606
Naïve Bayes	81.145	84.882	85.912	80.123	80.128
KNN	91.111	90.952	80.888	89.560	89.620
Proposed CCPM (Random Forest)	97.228	97.428	97.989	97.715	97.716

**Table 20:** Performance evaluation results based on iForest and SMOTE for Cytology.

Method	Precision (%)	Recall/Sensitivity (%)	Specificity (%)	F1 Score (%)	Accuracy (%)
SVM	94.333	97.963	90.293	94.041	94.043
MLP	93.771	94.291	93.677	91.774	91.724
Regression	83.098	79.487	87.980	84.671	84.641
Naïve Bayes	81.635	77.830	85.106	81.261	81.250
KNN	89.497	97.129	78.971	88.242	88.366
Proposed CCPM (Random Forest)	97.518	97.448	97.584	97.518	97.514

**Table 21:** Performance evaluation results based on iForest and SMOTETomek for Cytology.

Method	Precision (%)	Recall/Sensitivity (%)	Specificity (%)	F1 Score (%)	Accuracy (%)
SVM	93.231	99.038	85.556	92.512	92.537
MLP	91.998	92.788	94.913	92.523	92.234
Logistic Regression	85.784	77.669	92.422	84.093	84.900
Naïve Bayes	93.085	92.000	95.172	93.866	93.866
KNN	89.321	93.333	84.532	89.071	89.108
Proposed CCPM (Random Forest)	97.463	97.907	97.074	97.499	97.495

**Table 22:** Comparison of biopsy test results of CCPM with past studies.

Studies	Method	No of Features	Sensitivity (%)	Specificity (%)	Accuracy (%)
Wu and Zhu [10]	SVM-RFE	6	100	87.32	92.39
		18	100	90.05	94.03
	SVM-PCA	8	100	89.09	93.45
		11	100	90.05	94.03
Abdoh et al. [22]	Smote-RF-RFE	6	94.94	95.52	95.23
		18	94.42	97.26	95.87
	Smote-RF-PCA	8	93.77	97.26	95.55
		11	94.16	97.76	95.74
Present work	DBSCAN + SMOTETomek + RF	10	97.409	96.039	96.708
	DBSCAN + SMOTE+ RF	10	98.039	95.939	97.007
	iForest + SMOTETomek + RF	10	98.924	98.913	98.919
	iForest + SMOTE + RF	10	98.936	98.130	98.925

**Table 23:** Comparison of Schiller test results of CCPM with past studies.

Studies	Method	No of Features	Sensitivity (%)	Specificity (%)	Accuracy (%)
Wu and Zhu [10]	SVM-RFE	7	98.73	84.46	90.18
		18	98.73	84.63	90.18
	SVM-PCA	6	98.99	83.14	89.49
		12	98.99	84.30	90.18
Abdoh et al. [22]	Smote-RF-RFE	7	93.24	90.31	91.73
		18	93.51	92.35	92.91
	Smote-RF-PCA	6	92.70	96.17	94.49
		12	92.03	97.58	94.88
Present work	DBSCAN + SMOTETomek + RF	10	99.48	99.46	99.48
	DBSCAN + SMOTE+ RF	10	99.20	99.49	99.22
	iForest + SMOTETomek + RF	10	98.91	98.07	98.50
	iForest + SMOTE + RF	10	97.31	100	98.71

**Table 24:** Comparison of Hinselmann test results of CCPM with past studies.

Studies	Method	No of Features	Sensitivity (%)	Specificity (%)	Accuracy (%)
Wu and Zhu [10]	SVM-RFE	5	100	84.63	90.77
		15	100	84.49	93.69
	SVM-PCA	5	100	84.63	92.09
		11	100	84.65	93.79
Abdoh et al. [22]	Smote-RF-RFE	5	96.52	93.80	95.14
		15	96.65	95.14	95.88
	Smote-RF-PCA	5	96.52	98.30	97.42
		11	96.52	98.42	97.48
Present work	DBSCAN + SMOTETomek + RF	10	100	98.96	99.50
	DBSCAN + SMOTE+ RF	10	100	97.95	99.01
	iForest + SMOTETomek + RF	10	100	98.97	99.50
	iForest + SMOTE + RF	10	99.01	100	99.50

**Table 25:** Comparison of CYTOLOGY TEST results of CCPM with past studies.

Studies	Method	No of features	Sensitivity (%)	Specificity (%)	Accuracy (%)
Wu and Zhu [10]	SVM-RFE	8	100	84.42	90.65
		15	100	87.28	92.37
	SVM-PCA	8	100	86.65	91.98
		11	100	87.44	92.46
Abdoh et al. [22]	Smote-RF-RFE	8	87.37	97.54	92.52
		15	93.56	98.15	95.89
	Smote-RF-PCA	8	95.58	97.17	96.39
		11	95.32	98.40	96.89
Present work	DBSCAN + SMOTETomek + RF	10	97.43	98.01	97.72
	DBSCAN + SMOTE+ RF	10	96.43	98.01	97.22
	iForest + SMOTETomek + RF	10	97.91	97.08	97.50
	iForest + SMOTE + RF	10	97.45	97.58	97.51

**Table 26:** Time and Space Complexities of Machine Learning Algorithms.

Model Name	Time Complexity	Space Complexity
KNN	*O(knd)*	*O(nd)*
Logistic Regression	*O(nd)*	*O(d)*
SVM	O(*n²*)	O(*kd*)
Naive Bayes	O(*nd*)	O(*cd*)
Random Forest	O(*n*log(*n*)*dk*)	O(*depth of tree ∗ k*)

## Data Availability

The data used in present study is publicly available at the given link by UCI machine leaning repository. https://archive.ics.uci.edu/ml/datasets/Cervical+cancer+%28Risk+Factors%29.
